# Management of Leakage After Sleeve Gastrectomy: Outcome, Treatment Algorithm, and Predictors of Resolution

**DOI:** 10.7759/cureus.99914

**Published:** 2025-12-23

**Authors:** Mohamed A Salman, Adam Alguidi, Ahmed F Omar, Mohannad A Fayed, Ahmed Saeed H Saqr, Mai Alsadat, Ahmed Abdalla

**Affiliations:** 1 General Surgery, Kasralainy School of Medicine, Cairo, EGY; 2 General Surgery, University Hospital Ayr, Ayr, GBR; 3 General Surgery, Kasralainy School of Medicine, Cairo University, Cairo, EGY

**Keywords:** leakage, outcome, sleeve gastrectomy, treatment algorithm, upper gastrointestinal surgery

## Abstract

Background

Laparoscopic sleeve gastrectomy (LSG), which is increasing in popularity, is associated with certain complications. One of the most dreaded complications following LSG is a leakage from the staple line. Therefore, it is mandatory for surgeons to be alert to the risk factors of leakage and to be familiar with the leakage treatment choices. The aim of this study is to assess the post-LSG leakage rate, predictors of leakage, and present the treatment algorithm and its outcomes.

Methods

This retrospective cohort study included patients who underwent LSG at our institution during the period from 2018 to 2023. The diagnosis and management algorithm of leakage, as well as the outcomes of treatment, were assessed.

Results

Out of the included 1289 patients, leakage occurred in 14 patients (1.09%). All patients with leakage primarily received supporting treatment. Finally, the leak resolution rate was 92.86% (13/14). Leakage-management complications occurred in four patients (28.57%), all of whom required ICU admission. Regression analysis showed that higher BMI was the only significant predictor of non-resolution after initial treatment (p<0.001), while smoking (p = 0.002) and diabetes mellitus (p = 0.009) were the predictors of leakage occurrence.

Conclusion

The treatment algorithm used in the current study proved effective in managing post-LSG leakage, despite the occurrence of some complications. Computed tomography (CT) and endoscopy demonstrated a complementary diagnostic role in accurately identifying leaks. Smoking and diabetes mellitus were identified as predictors of leakage occurrence, while higher BMI was significantly associated with non-resolution of leakage after initial treatment.

## Introduction

Several bariatric options have existed as a long-term treatment for severe obesity, which has become a worldwide pandemic. Among them is laparoscopic sleeve gastrectomy (LSG), which has gained increasing popularity due to its simplicity and the growing worldwide evidence supporting its efficacy and safety [1,2]. However, as with any major surgery, LSG has its associated complications that occur at a rate ranging from about 1.2% up to 7.3% [3,4].

One of the most dreaded complications following LSG is a leakage from the staple line that is encountered in about 0.7% to 5% of patients [5]. Post-LSG leak has been described as a main cause of prolonged length of stay (LOS) in hospitals and mortality [6].

Therefore, it is mandatory for surgeons, particularly with the daily increase in the number of performed LSG, to be alert to the risk factors and early signs warranting leakage occurrence and to be familiar with the leakage treatment choices.

Variable leakage treatment algorithms have been proposed in the literature. However, these algorithms were grounded on diverse methodologies shaped by the financial conditions of each country and the absence of a global consensus [5,7-12]. As a result, the approach to leakage treatment often depends heavily on the surgeon's preference and the available financial resources, leading to significant variability in outcomes. Additionally, scarce evidence is available regarding the predictors of leakage outcomes [12,13]. This topic has been scarcely explored in the Egyptian setting. The aim of this study is to assess the post-LSG leakage rate and the predictors of leakage, as well as to present the treatment algorithm and its outcomes in an Egyptian population.

## Materials and methods

This is a retrospective analysis of prospectively collected data of patients who underwent LSG at our institution during the period from 2018 to 2023. The study was conducted per the Declaration of Helsinki after being approved by the research ethics committee.

Patients’ eligibility for bariatric surgery at our institution was based on the 2022 American Society for Metabolic and Bariatric Surgery-International Federation for the Surgery of Obesity and Metabolic Disorders (ASMBS-IFSO) indications for metabolic and bariatric surgery, as published in their joint guideline [14]. Adult patients who underwent LSG during the period from 2018 to 2023 were included. Follow-up duration was based on the routine postoperative schedule used at our institution, which typically includes visits at one week, one month, three months, six months, and 12 months, with additional visits as clinically indicated. As this was a retrospective file-based study, completeness of follow-up was ensured by reviewing the entire electronic medical record for each patient, including outpatient notes, emergency visits, inpatient admissions, radiological reports, and laboratory results, and excluding any patients with missing or insufficient follow-up documentation. Patients who had had previous bariatric procedures were also excluded from the study.

Data regarding the patients’ preoperative personal and medical history, clinical data, and radiological assessment, as well as the perioperative data and the surgery outcomes, were screened, recorded, and analyzed.

Diagnosis and management of leakage

Patients with a clinically suspected leak underwent an abdominal computed tomography (CT) scan with water-soluble oral and IV contrast after proper preparation. CT images were analyzed to identify the leak and evaluate surrounding structures, and all scans were interpreted by an experienced gastrointestinal radiologist. Leakage diagnosis was considered in the presence of intra-abdominal fluid collection, perigastric inflammation signs such as fat stranding and perigastric fluid/air locules, or contrast extravasation. Endoscopic evaluation was performed for all patients with clinical suspicion of a leak, even if the CT findings were equivocal, as endoscopy provided direct visualization that allowed precise localization of the leak and assessment of mucosal characteristics that were not fully determined by CT.

Gastric leaks were categorized as acute (leakage diagnosed within one week of surgery), early (leakage diagnosed within one to six weeks), late (leakage diagnosed within six to 12 weeks), and chronic (leakage diagnosed 12 weeks or beyond) [15].

Patients with leakage were initially treated with nothing per os (NPO) and intravenous nutritional support. Proton pump inhibitors (PPIs) and broad-spectrum antibiotics were administered as part of the initial management. PPIs were given intravenously (e.g., pantoprazole 40 mg once daily), and broad-spectrum antibiotics were initiated according to our institutional protocol (typically a combination such as piperacillin-tazobactam 4.5 g IV every six hours), with adjustments based on culture results and clinical response. Hemodynamically unstable patients and patients with signs of generalized peritonitis were subjected to laparoscopic lavage and drainage before managing the leakage site. In hemodynamically stable patients, if the fluid collection was shown to be feasibly drained by percutaneous drainage, CT-guided drainage was performed.

Two endoscopic techniques were then used for the treatment of leakage: endoscopic internal drainage (EID) with pigtail catheters and stenting. Double pigtail catheters were used in cases of a single opening less than 5 mm, allowing drainage of fluid from the leak site. Otherwise, endoscopic stenting was done. Covered self-expanding metal stents (SEMS) were used. After the endoscopic introduction of the stent, it expanded to cover and seal the leak. Some surgeons used clipping to secure the stent in place.

Study outcomes

The primary outcomes of the current study were the incidence of post-LSG leak occurrence, the rate of resolution of the treated leakage, associated complications, LOS, ICU admission, and mortality rate. The secondary outcomes were the potential predictors/risk factors of post-LSG leak and the predictors of leakage resolution.

Statistical methods

Analysis of the collected patients’ data was done using Jamovi (Jamovi, Version 2.3, Computer Software, Retrieved from https://www.jamovi.org, Sydney, Australia). Data were expressed as range and mean ± standard deviation (SD) if numerical, and number (percentage) if categorical. Binary logistic regression analysis was conducted to determine the predictors/risk factors of leakage occurrence and resolution after treatment. Statistical significance was set at 0.05.

## Results

This study included 1,289 patients who underwent LSG during the study period. The patients’ age ranged from 18 to 65 years, with a mean of 38.04 ± 6.865 years. Most patients were females (n = 978; 75.9%). The patients’ baseline BMI ranged from 39.8 to 60.2 kg/m2, with a mean value of 42.06 ± 3.44 kg/m^2^. Smoking was prevalent in 157 patients (12.18%). The patients’ obesity-associated complications were dyslipidemia (n = 1057; 82%), hypertension (n = 466; 36.15%), type 2 diabetes mellitus (n = 243; 18.85%), and obstructive sleep apnea (n = 241; 18.7%) (Table 1).

**Table 1:** The baseline characteristics of the total cohort (n=1289) and the leakage group (n=14)

Variable	Total Cohort (n=1289)	Leakage Group (n=14)
Age (years), Mean ± SD	38.04±6.865	40.11±10.15
Female Gender, n (%)	978 (75.9%)	10 (71.43%)
BMI (kg/m²), Mean ± SD	42.06±3.44	52.43±5.6
Smoking Status, n (%)	157 (12.18%)	4 (28.57%)
Hypertension, n (%)	466 (36.15%)	7 (50.0%)
Diabetes Mellitus, n (%)	243 (18.85%)	6 (42.86%)
Leakage Occurrence, n (%)	14 (1.09%)	-

Out of the included 1,289 patients, leakage occurred in 14 patients (1.09%). Their age ranged from 21 to 60 years, with a mean of 40.11 ± 10.15 years and a gender predilection to females (n = 10; 71.43%). The BMI ranged from 43.23 kg/m^2^ to 60.07 kg/m^2^, with a mean of 52.43 ± 5.6 kg/m^2^. Smoking was prevalent in four patients (28.57%). The obesity-associated medical complications were dyslipidemia (n = 11; 78.57%), hypertension (n = 7; 50.0%), type 2 diabetes mellitus (n = 6; 42.86%), and obstructive sleep apnea (n = 2; 14.29%) (Table 2).

**Table 2:** Characteristics of patients with leakage (n = 14) LOS: length of stay; EID: endoscopic internal drainage

Variable	Measure	Value (n=14)
Leakage Incidence, n (%)	Overall	14 (1.09%)
Time to Leakage (days)	Mean ± SD (Range)	15.64±6.69 (5−29)
Leakage Timing, n (%)	Early leaks (>7–29 days)	11 (78.57%)
Key Presenting Symptoms, n (%):	Abdominal pain	14 (100%)
	Tachycardia	12 (85.71%)
Key CT Finding, n (%)	Intra-abdominal collection	12 (85.71%)
Urgent Surgery Required, n (%)		7 (50.0%)
Final Resolution Rate, n (%)		13/14 (92.86%)
Overall LOS (Days)	Mean ± SD (Range)	21.29±12.75 (5−45)
LOS by Primary Management Method (Mean ± SD)	CT-Guided Drainage	14.5±5.0 Days
	EID (Small Fistula)	18.0±6.0 Days
	Endoscopic Stenting (Large Fistula)	28.0±8.0 Days
ICU Admission, n (%)		4 (28.57%)
ICU LOS (days)	Mean ± SD	8.5±3.5
Mortality, n (%)		1 (7.14%)

In patients with leakage, the time interval from surgery to presentation with leakage ranged from five to 29 days, with a mean of 15.64 ± 6.69 days. According to the time of presentation, three patients (21.43%) had acute leaks (from five to seven days after surgery), and the remaining 11 patients (78.57%) had early leaks (from more than seven days to 29 days after surgery) (Table 2).

The patients with leaks mainly presented with abdominal pain (n = 14; 100%), tachycardia (n = 12; 85.71%), fever (n = 9; 64.29%), vomiting (n = 6; 42.86%), and nausea (n = 6; 42.86%). Other symptoms were drowsiness (n = 2; 14.29%), tachypnea (n = 2; 14.29%), chills (n = 2; 14.29%), abdominal distention (n = 1; 7.14%), cough (n = 1; 7.14%), and port-site leakage (n = 1; 7.14%) (Table 2).

Abdominal CT examination revealed intra-abdominal collection in 12 patients (85.71%), with 10 patients (71.43%) demonstrating contrast extravasation, while two patients (14.29%) had uncertain CT results. Endoscopic assessment revealed a small fistulous opening in six patients (42.86%), a large fistulous opening in six patients (42.86%), and a suspicious erythematous area in two patients (14.29%) (Table 2).

**Figure 1:**
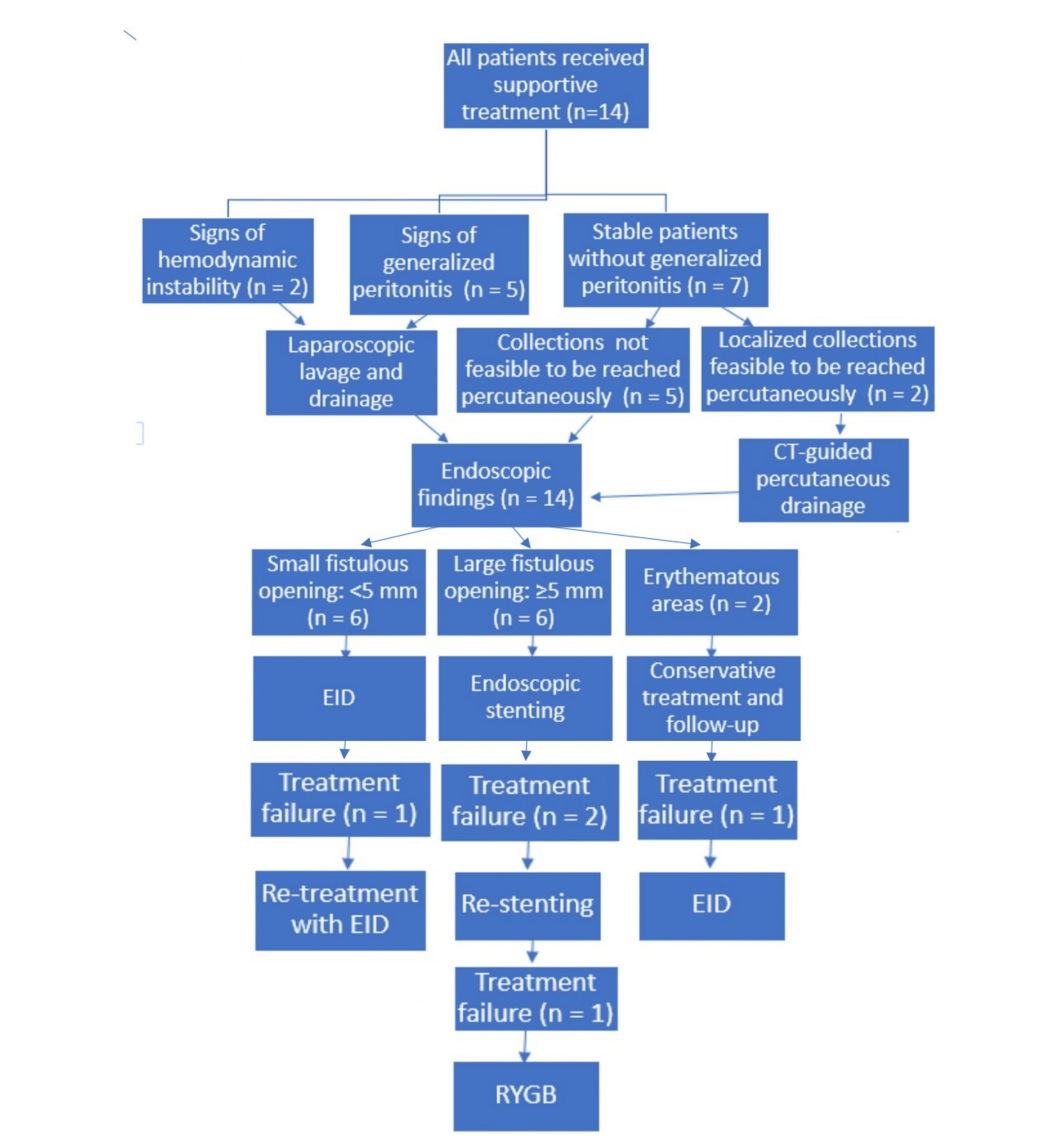
Treatment algorithm utilised in patient care EID: endoscopic internal drainage; RYGB: Roux-en-Y gastric bypass

The patients’ treatment algorithm is demonstrated in Figure 1. All patients with leakage primarily received NPO, nutritional support, PPIs, and antibiotics. Two patients had localized collections feasible to be reached percutaneously and underwent CT-guided drainage. Seven patients (50.0%) required urgent surgical intervention due to signs of peritonitis (n = 5; 35.71%) or hemodynamic instability (n = 2; 14.29%).

Patients found to have small fistulous openings by endoscopy (n = 6; 42.86%) were treated with EID using double pigtail catheters, and those with large fistulous openings (n = 6; 42.86%) were managed with stent placement. Patients with uncertain CT findings and endoscopically detected erythema were followed up after conservative treatment. Patients' feeding was through total parenteral nutrition (n = 10; 71.43%), nasojejunal tube (n = 2; 14.29%), feeding jejunostomy (n = 1; 7.14%), and oral feeding (n = 1; 7.14%).

Re-leak/persistent leak occurred in four patients (28.57%): one from patients treated with EID and had a double-pigtail catheter re-inserted, two from patients treated with stenting and were re-stented, and one from patients treated conservatively and underwent EID.

A persistent leak was encountered in one of the re-stented patients. The patient finally converted to Roux-en-Y gastric bypass (RYGB). Finally, the leak resolution rate was 92.86% (13/14).

The leakage management complications occurred in four patients (28.57%). One patient (7.14%) had an esophagobronchial fistula that was complicated with septic shock and pneumonia and then was further complicated by pulmonary embolism; one patient (7.14%) had a presplenic abscess; one patient (7.14%) had a gastrocutaneous fistula; and the last patient (7.14%) had pneumonia and septic shock.

The patients' LOS ranged from five to 45 days, with a mean of 21.29 ± 12.75 days. ICU admission was required in four patients with complications (28.57%). Mortality was encountered in one patient (7.14%) who had pulmonary embolism.

Univariate binary logistic regression analysis of the baseline patients' criteria revealed that smoking (β = 1.734, 95% CI: 1.255 to 2.221, OR = 5.662, p = 0.002) and the presence of diabetes mellitus (β = 1.459, 95% CI: 1.103 to 1.964, OR = 4.302, p = 0.009) were the predictors of leakage occurrence (Table 3), while the patients' BMI (β = 2.014, 95% CI: 1.567 to 2.562, OR = 7.491, p < 0.001) was the significant predictor of non-resolution of leak after the initial treatment (Table 4).

**Table 3:** Univariate binary logistic regression for predictors of leakage occurrence

Variable	Beta (β)-Coefficient	Odds Ratio (OR)	95% CI	p-value
Age	0.021	1.02	0.97 – 1.07	0.412
Gender (Female)	0.284	1.33	0.41 – 4.25	0.632
BMI	0.067	1.07	0.92 – 1.24	0.337
Smoking	1.734	5.662	1.255 – 2.221	0.002
Dyslipidemia	0.194	1.21	0.35 – 4.22	0.764
Hypertension	0.381	1.46	0.48 – 4.37	0.497
Diabetes Mellitus	1.459	4.302	1.103 – 1.964	0.009
Obstructive Sleep Apnea	0.327	1.39	0.29 – 6.60	0.681

**Table 4:** Univariate binary logistic regression for predictors of non-resolution of the leak

Variable	Beta (β)-Coefficient	Odds Ratio (OR)	95% CI	p-value
Age	0.044	1.04	0.92 – 1.18	0.512
Gender (Female)	0.263	1.30	0.18 – 9.33	0.789
Smoking	0.381	1.46	0.20 – 10.42	0.702
Diabetes Mellitus	0.492	1.64	0.27 – 9.78	0.595
Hypertension	0.214	1.24	0.21 – 7.40	0.822
Dyslipidemia	0.154	1.17	0.12 – 10.69	0.894
Obstructive Sleep Apnea	0.317	1.37	0.12 – 15.45	0.792
BMI	2.014	7.491	1.567 – 2.562	<0.001

## Discussion

Despite the well-established advantages of LSG as one of the most effective and safe bariatric choices, LSG is not devoid of complications. One of the most formidable among these is staple line leakage, contributing substantially to surgery-associated morbidity and mortality. Given the increasing volume of this procedure worldwide, it is imperative to identify patients at risk and be well-versed in the spectrum of available treatment modalities. Determination of factors that are risky for the leak occurrence and non- or delayed resolution of leakage can enrich surgical practice and perioperative care strategy, eventually improving patient safety and treatment efficacy.

This study is particularly significant as it addresses these gaps within the context of the Egyptian healthcare system, a setting that has not been extensively explored in previous research. By examining the incidence, management strategies, and outcomes of staple line leakage post LSG in Egypt, the study contributes to the broader body of knowledge on this subject.

The current study finding, where a leakage rate of 1.09% was observed among 1,289 patients who underwent LSG, aligns with the literature-reported post-LSG leakage rates. In close proximity to our data, Shawabkah et al. [16] found a leakage rate of about 1.5% in a cohort of 400 patients. Alzahrani et al. [17] in a Saudi tertiary center reported a lower leakage rate of 0.53%. On the other hand, higher rates were found by Gagner and Buchwald [18], Cesana et al. [19], and Ser et al. [20], who reported leakage rates of 2.1%, 2.6%, and 3.39%, respectively. The study by Verras et al. [21] reported a notably higher leakage rate of 6.46%. This variation in the leakage rate suggests differences in patient selection, surgical expertise, or postoperative monitoring practices.

In the context of confirming post-LSG leakage in patients with clinical suspicion, the present study emphasizes that CT assessment and endoscopy use play a complementary role. While CT helped to identify intra-abdominal collections and confirm leakage, endoscopy provided a detailed assessment of the mucosal integrity and the extent of fistulous openings and identified leakage when CT findings were equivocal. Nitin et al. [22] and Manos et al. [7] further supported this by demonstrating the effectiveness of endoscopy in both diagnosis and management of leakage, particularly when a CT scan is inconclusive. Combining these modalities ensures that even subtle leakage, which could lead to severe complications if undetected, is identified and appropriately managed, thus improving patient outcomes in post-LSG care.

The current study treatment algorithm, where the patients’ clinical conditions, CT findings, and endoscopic results tailored the selected treatment approach that varied from conservative management to endoscopic procedures and surgical interventions, aligns with and contrasts with other proposed algorithms in previous studies. Manos et al. [7] emphasized using septotomy with balloon dilatation and pigtail insertions for endoscopic management. This was not addressed in the present work because no cases of stenosis were encountered since all cases had either acute or early leaks. Bashah et al. [12] proposed a multimodal approach in which early surgical exploration was limited to cases with active contrast extravasation in CT assessment. However, like our study, they treated small fistulas with EID and large ones with stenting. Nimeri et al. [5], similar to this study, advocated for early surgical exploration in the presence of clinical peritonitis, but their protocol includes intraoperative endoscopy and placement of a jejunostomy tube. Nimeri et al. [5], on the other hand, reserved stent placement for patients who are stable and without strictures. For difficult cases not amenable to stenting, Nimeri et al. [5] recommend laparoscopic Roux-en-Y esophagojejunostomy.

Overall, comparing our results to other published algorithms revealed that, whereas there are common areas in the strategy of management, the particular interventions varied based on the clinical scenario. This highlights the need for a tailored approach that considers both the timing of the leak, the patient's clinical state, and the available familiar interventional methods.

In this study, leakage management showed a complication rate of 28.57% and an ICU admission and mortality rate of 7.14%. In line with our study, Alzahrani et al. [17] reported a mortality rate of 12.5% and described treatment-related complications, including esophageal stenosis and stent migration. Contrasting this, Bashah et al. [12] reported no mortality among 73 leakage cases. Variation in outcome is likely attributable to the variation in clinical severity and types of complications.

Finally, the overall success rate of the current study was 92.86%. Likewise, Manos et al. [7], Bashah et al. [12], and Rebibo et al. [13] reported healing rates of 96.2%, 97.1%, and 93.5%, respectively. A higher rate was achieved by Currò et al. (2018), who presented a 100% success rate in eight patients with leakage [23]. Lower healing rates were observed by Alzahrani et al. [17], and Li et al. [24] observed resolution rates of 88.9% and 87.5%, respectively. These differences in healing rates reflect variations in patients’ criteria, leakage onset, and treatment algorithms.

The present study illustrated smoking and diabetes mellitus as risk factors for leakage occurrence. In alignment with our findings, Głuszyńska et al. [25] reported smoking as an independent predictor of LSG complications, with a particular risk for post-LSG leakage. Diabetes mellitus was identified by Verras et al. [21] as a risk factor for post-LSG leakage. This can be explained by the impact of smoking on microvascular circulation, which is crucial for tissue healing. Despite the different pathophysiological mechanisms, diabetes mellitus also adversely affects the microvascular circulation, with subsequent impaired wound healing. Furthermore, it increases the risk of infection, which hinders tissue healing.

As for the non-resolution of leakage, higher BMI was identified as a risk factor in this study. Supporting our findings, Bracale et al. [26], in their recent systematic review, found that higher BMI was a critical risk factor for anastomotic leakage after LSG. This may be related to the greater technical challenges often facing patients with a higher BMI during surgery. This was also found by He et al. [27], who reported higher BMI as a risk factor for poor outcomes after leakage treatment.

This study contributes to the ongoing discourse on optimizing LSG outcomes, supporting the individualized care protocols that consider clinical scenarios. The study is limited by its retrospective design and the relatively limited number of patients with leakage. Also, this study did not include patients with chronic leaks (>30 days), and therefore, the findings primarily apply to acute and early postoperative leaks. Additionally, the timing of stent removal was not fully standardized across all cases, which may introduce variability in the treatment course and outcome assessment. These factors should be considered when interpreting the results. Moreover, this study was conducted at a single, high-volume bariatric center, and therefore, the findings, especially the low leak incidence, may reflect institutional practices and may not be fully generalizable to all settings. Although the overall sample is large, the number of leak events is very small (n=14), which severely limits the statistical power of the regression analysis. Because the number of outcomes is far below the recommended threshold for reliable multivariable modeling, the identified predictors (smoking, diabetes mellitus, and BMI for non-resolution) must be interpreted as exploratory associations rather than definitive risk factors. These results may be affected by overfitting and should be validated in larger cohorts.

## Conclusions

While our algorithm achieved a high final resolution rate, it was associated with significant morbidity and a high need for re-intervention. CT and endoscopy demonstrated a complementary diagnostic role in accurately identifying leaks. Smoking and diabetes were associated with leaks in our small cohort, but this finding is severely limited by low statistical power. Smoking and diabetes mellitus were identified as predictors of leakage occurrence. Future multicenter prospective studies with larger cohorts are recommended to validate these predictive factors and to optimize the treatment algorithm for postoperative leaks following LSG.

## References

[1] (2023). 7th IFSO global registry report. https://www.ifso.com/pdf/ifso-7th-registry-report-2022.pdf.

[2] Angrisani L, Santonicola A, Iovino P, Ramos A, Shikora S, Kow L (2021). Bariatric surgery survey 2018: similarities and disparities among the 5 IFSO chapters. Obes Surg.

[3] Guetta O, Vakhrushev A, Dukhno O, Ovnat A, Sebbag G (2019). New results on the safety of laparoscopic sleeve gastrectomy bariatric procedure for type 2 diabetes patients. World J Diabetes.

[4] Hutter MM, Schirmer BD, Jones DB, Ko CY, Cohen ME, Merkow RP, Nguyen NT (2011). First report from the American College of Surgeons Bariatric Surgery Center Network: laparoscopic sleeve gastrectomy has morbidity and effectiveness positioned between the band and the bypass. Ann Surg.

[5] Nimeri A, Ibrahim M, Maasher A, Al Hadad M (2016). Management algorithm for leaks following laparoscopic sleeve gastrectomy. Obes Surg.

[6] Aurora AR, Khaitan L, Saber AA (2012). Sleeve gastrectomy and the risk of leak: a systematic analysis of 4,888 patients. Surg Endosc.

[7] Manos T, Nedelcu M, Nedelcu A (2021). Leak after sleeve gastrectomy: updated algorithm of treatment. Obes Surg.

[8] Abou Rached A, Basile M, El Masri H (2014). Gastric leaks post sleeve gastrectomy: review of its prevention and management. World J Gastroenterol.

[9] Gagner M (2014). Decreased incidence of leaks after sleeve gastrectomy and improved treatments. Surg Obes Relat Dis.

[10] Parmer M, Wang YH, Hersh EH, Zhang L, Chin E, Nguyen SQ (2022). Management of staple line leaks after laparoscopic sleeve gastrectomy. JSLS.

[11] Alizadeh RF, Li S, Inaba C (2018). Risk factors for gastrointestinal leak after bariatric surgery: MBASQIP analysis. J Am Coll Surg.

[12] Bashah M, Khidir N, El-Matbouly M (2020). Management of leak after sleeve gastrectomy: outcomes of 73 cases, treatment algorithm and predictors of resolution. Obes Surg.

[13] Rebibo L, Tricot M, Dembinski J, Dhahri A, Brazier F, Regimbeau JM (2021). Gastric leak after sleeve gastrectomy: risk factors for poor evolution under conservative management. Surg Obes Relat Dis.

[14] Eisenberg D, Shikora SA, Aarts E (2023). Society for Metabolic and Bariatric Surgery (ASMBS) and International Federation for the Surgery of Obesity and Metabolic Disorders (IFSO): indications for metabolic and bariatric surgery. Obes Surg.

[15] Rosenthal RJ, Diaz AA, Arvidsson D (2012). International Sleeve Gastrectomy Expert Panel consensus statement: best practice guidelines based on experience of >12,000 cases. Surg Obes Relat Dis.

[16] Shawabkah O, Atyiat M, Aladwan K, Alshahwan R, Khraisat H, Alduham M (2023). Frequency of leakage after surgery following leak test during laparoscopic sleeve gastrectomy.. Scholars J Appl Med Sci.

[17] Alzahrani K, Abdelrahman T, Jafri S, Abdo M, Alsharif K (2022). Leakage management after laparoscopic sleeve gastrectomy: a tertiary center experience in Saudi Arabia.. World Fam Med J.

[18] Gagner M, Buchwald JN (2014). Comparison of laparoscopic sleeve gastrectomy leak rates in four staple-line reinforcement options: a systematic review. Surg Obes Relat Dis.

[19] Cesana G, Cioffi S, Giorgi R (2018). Proximal leakage after laparoscopic sleeve gastrectomy: an analysis of preoperative and operative predictors on 1738 consecutive procedures. Obes Surg.

[20] Ser KH, Lee WJ, Lee YC, Chen JC, Su YH, Chen SC (2010). Experience in laparoscopic sleeve gastrectomy for morbidly obese Taiwanese: staple-line reinforcement is important for preventing leakage. Surg Endosc.

[21] Verras GI, Mulita F, Lampropoulos C (2023). Risk factors and management approaches for staple line leaks following sleeve gastrectomy: a single-center retrospective study of 402 patients. J Pers Med.

[22] Nitin J, Yashavanth HS, Kalapala R (2020). Endoscopic management of postlaparoscopic sleeve gastrectomy leaks: a single-center experience. J Dig Endosc.

[23] Li M, Zeng N, Liu Y (2023). Management and outcomes of gastric leak after sleeve gastrectomy: results from the 2010-2020 national registry. Chin Med J (Engl).

[24] Currò G, Komaei I, Lazzara C, Sarra F, Cogliandolo A, Latteri S, Navarra G (2018). Management of staple line leaks following laparoscopic sleeve gastrectomy for morbid obesity. Surg Technol Int.

[25] Głuszyńska P, Diemieszczyk I, Szczerbiński Ł, Krętowski A, Major P, Razak Hady H (2022). Risk factors for early and late complications after laparoscopic sleeve gastrectomy in one-year observation. J Clin Med.

[26] Bracale U, Peltrini R, De Luca M (2022). Predictive factors for anastomotic leakage after laparoscopic and open total gastrectomy: a systematic review. J Clin Med.

[27] He Z, Liu H, Zhou L (2023). Risk factors and conservative therapy outcomes of anastomotic leakage after gastrectomy: experience of 3,926 patients from a single gastric surgical unit. Front Oncol.

